# Alterations of Serum Magnesium Concentration in Animal Models of Seizures and Epilepsy—The Effects of Treatment with a GPR39 Agonist and Knockout of the *Gpr39* Gene

**DOI:** 10.3390/cells11131987

**Published:** 2022-06-21

**Authors:** Urszula Doboszewska, Jan Sawicki, Adam Sajnóg, Aleksandra Szopa, Anna Serefko, Katarzyna Socała, Mateusz Pieróg, Dorota Nieoczym, Katarzyna Mlyniec, Gabriel Nowak, Danuta Barałkiewicz, Ireneusz Sowa, Piotr Wlaź

**Affiliations:** 1 Department of Animal Physiology and Pharmacology, Institute of Biological Sciences, Maria Curie-Skłodowska University, Akademicka 19, PL 20-033 Lublin, Poland; katarzyna.socala@mail.umcs.pl (K.S.); mateusz.pierog@mail.umcs.pl (M.P.); dorota.nieoczym@mail.umcs.pl (D.N.); piotr.wlaz@mail.umcs.pl (P.W.); 2 Department of Pharmacobiology, Jagiellonian University Medical College, Medyczna 9, PL 30-688 Kraków, Poland; katarzyna.mlyniec@uj.edu.pl (K.M.); nowak@if-pan.krakow.pl (G.N.); 3 Department of Analytical Chemistry, Medical University of Lublin, Chodźki 4A, PL 20-093 Lublin, Poland; jansawicki@umlub.pl (J.S.); i.sowa@umlub.pl (I.S.); 4 Department of Trace Analysis, Adam Mickiewicz University, Uniwersytetu Poznanskiego 8, PL 61-614 Poznan, Poland; adam.sajnog@amu.edu.pl (A.S.); danutaba@amu.edu.pl (D.B.); 5 Chair and Department of Applied and Social Pharmacy, Laboratory of Preclinical Testing, Medical University of Lublin, Chodźki 1, PL 20-093 Lublin, Poland; aleksandra.szopa@umlub.pl (A.S.); anna.serefko@umlub.pl (A.S.); 6 Department of Neurobiology, Maj Institute of Pharmacology, Polish Academy of Sciences, Smetna 12, PL 31-343 Kraków, Poland

**Keywords:** GPR39, TC-G 1008, zinc chloride, magnesium, seizures, epilepsy

## Abstract

Several ligands have been proposed for the GPR39 receptor, including the element zinc. The relationship between GPR39 and magnesium homeostasis has not yet been examined, nor has such a relationship in the context of seizures/epilepsy. We used samples from mice that were treated with an agonist of the GPR39 receptor (TC-G 1008) and underwent acute seizures (maximal electroshock (MES)- or 6-hertz-induced seizures) or a chronic, pentylenetetrazole (PTZ)-induced kindling model of epilepsy. MES seizures and PTZ kindling, unlike 6 Hz seizures, increased serum magnesium concentration. In turn, *Gpr39*-KO mice that underwent PTZ kindling displayed decreased concentrations of this element in serum, compared to WT mice subjected to this procedure. However, the levels of expression of TRPM7 and SlC41A1 proteins—which are responsible for magnesium transport into and out of cells, respectively—did not differ in the hippocampus between *Gpr39*-KO and WT mice. Furthermore, laser ablation inductively coupled plasma mass spectrometry applied to hippocampal slices did not reveal differences in magnesium levels between the groups. These data show the relationship between magnesium homeostasis and certain types of acute or chronic seizures (MES seizures or PTZ kindling, respectively), but do not explicitly support the role of GPR39 in mediating magnesium balance in the hippocampus in the latter model. However, decreased expression of TRPM7 and increased expression of SLC41A1—which were observed in the hippocampi of *Gpr39*-KO mice treated with TC-G 1008, in comparison to WT mice that received the same treatment—implicitly support the link between GPR39 and hippocampal magnesium homeostasis.

## 1. Introduction

About one-third of human GPCRs are orphan receptors, for which endogenous ligands are unknown [1]. Characterization of orphan receptors may lead to uncovering physiological functions, thus connecting them to diseases. GPR39 is among the class A orphan receptors [2]. Several endogenous agonists have been proposed, including the element zinc [3,4], yet the classification of GPR39 as a zinc receptor remains contentious. Other metal ions, such as nickel [4], cadmium, chromium, and iron ions [5], have also been reported to activate GPR39. The relationship between GPR39 and magnesium has not been examined.

GPR39 is being investigated in relation to cardiovascular diseases [6], neurovascular diseases [7], diseases of the central nervous system (CNS) such as depression [8,9] or epilepsy [10,11], bone diseases [12], etc. Both elements—zinc and magnesium—play significant roles in the aforementioned conditions, more specifically in cardiovascular diseases [13,14], depression [15], epilepsy [16], or diseases of bones [17,18,19]. There is an overlap between the actions of these elements at the level of biochemistry/pharmacology. They share a common gatekeeper—the transient receptor potential (TRP) channel, subfamily M, member 7 (TRPM7). In the intestines, TRPM7 is the master regulator of magnesium and zinc balance [20]. Likely, in the brain, TRPM7 is important for the homeostasis of these divalent cations [21]. Furthermore, zinc and magnesium modulate the function of TRP channels [22] and other receptors [23] whose role is implicated in the abovementioned pathological states [24].

In this study we used samples obtained from mice that were subjected to models of acute seizures or a chronic model of epilepsy, as an example of a research area in which GPR39 is being investigated. A link between GPR39 and seizures/epilepsy has been suggested in a few studies. The expression of GPR39 at either the protein or RNA levels was altered in animal models—in the hippocampi of mice [25] or in zebrafish [26]. *Gpr39*-knockout (KO) mice exhibited enhanced susceptibility to acute seizures induced by a chemical substance—kainic acid (KA) [10,27]. We compared the behavior of *Gpr39*-KO and wild-type (WT) mice in another model of acute seizures, induced by an electrical stimulus—namely, the maximal electroshock seizure (MES) model. Moreover, we employed a chronic, pentylenetetrazole (PTZ)-induced kindling model of epilepsy, which reflects the process of epilepsy’s development, i.e., epileptogenesis. In our hands, the genotype had no effect on the seizure threshold in response to MES, or on epileptogenesis induced by PTZ [11]. It is noteworthy that we observed decreased serum zinc concentrations in *Gpr39*-KO mice subjected to the PTZ-kindling model, compared to WT mice that underwent this model [11], thus adding data supporting the link between the GPR39 receptor and zinc. 

Furthermore, by combining the observations of the behavioral effects of the GPR39 agonist TC-G 1008 (compound 3 [28], GPR39-C3 [29]) in genetically unmodified mice and in *Gpr39*-KO mice, we found that TC-G 1008 facilitated PTZ-induced epileptogenesis by acting at the GPR39 receptor. Taken together, the genotype per se did not affect PTZ-induced epileptogenesis, but activation of GPR39 by TC-G 1008 aggravated epileptogenesis in this model. In acute seizure tests, TC-G 1008 exerted divergent effects. It decreased the seizure threshold in the maximal electroshock seizure threshold (MEST) test, while it increased the seizure threshold in the 6-hertz-induced seizure threshold test [11].

In addition, clinical data showed the importance of monitoring magnesium levels in those with neurological conditions [30]. Variants of the *TRPM6* gene—another TRP channel belonging to subfamily M, which is a close homolog of TRPM7 [31]—caused hypomagnesaemia accompanied by seizures [32]. Two recent studies did not find seizures as a significant risk factor for hypomagnesaemia [33,34], but seizures are among the neurological symptoms that may occur in those with severe hypomagnesaemia [35,36]. A meta-analysis of 40 studies showed that the serum magnesium concentrations were not different between epilepsy patients and control subjects, but hair magnesium concentrations were lower in both treated and non-treated patients compared to controls. Moreover, serum magnesium concentrations were increased in epileptic patients on valproate (VPA) monotherapy compared to untreated patients, while they were not different compared to control subjects [37]. These data warrant further examination of associations between epilepsy and magnesium homeostasis.

We hypothesized that there might be an interaction between GPR39 and magnesium in the context of seizures/epileptogenesis. To preliminary explore this possible relationship, we measured magnesium concentrations in serum samples of mice subjected to models of acute seizures or the PTZ kindling model. We also examined serum concentrations of this element in *Gpr39*-KO mice subjected to the PTZ kindling model. Decreased magnesium concentrations in the sera of *Gpr39*-KO mice led us to investigate whether there were accompanying alterations in the brain. Using the methodology presented here we did not, however, demonstrate corresponding changes in the hippocampal tissue. More surprisingly, the GPR39 agonist decreased the expression of the TRPM7 protein in the hippocampus of *Gpr39*-KO mice, thus adding data suggestive of the non-selective activity of this compound [11,29].

## 2. Materials and Methods

### 2.1. Animals

The experiments were performed in genetically unmodified mice (Swiss albino mice) and in genetically modified mice bred with a mixed genetic background (C57BL/6/Tar × CBA/Tar mice). Housing and experimental procedures were conducted in accordance with the European Union Directive of 22 September 2010 (2010/63/EU), as well as Polish legislation acts concerning animal experimentation. The procedures were approved by the Local Ethics Committee in Lublin (experiments in genetically unmodified mice: approval numbers 38/2017, 48/2018, and 36/2019; experiments in *Gpr39*-KO mice: approval numbers 72/2019 and 16/2020) and the I Local Ethics Committee in Warsaw (approval number 811/2019 regarding generation of the *Gpr39*-KO mouse line).

Experimentally naïve male Swiss albino mice (*n* = 278) with a body weight range of 17–31 g were purchased from a licensed breeder (Laboratory Animals Breeding, Ilkowice, Poland). They were housed in groups of 7–8 in open Makrolon cages (37 cm × 21 cm × 14 cm) under strictly controlled laboratory conditions (temperature maintained at 21–24 °C, relative humidity at 45–65%) with an artificial 12/12 h light/dark regime (light on at 6:00 a.m.). A rodent chow diet (Murigran, Agropol S.J., Motycz, Poland) and tap water were provided ad libitum. The environment was enriched with nest material and paper tubes.

The *Gpr39*-KO mouse model was generated by the Mouse Genome Engineering Facility (crisprmice.eu, accessed on 31 December 2019). A CRISPR/Cas9 method was used to establish the model with a mixed genetic background (C57BL/6/Tar × CBA/Tar). A deletion of 44 bp causing a p.Lys38fs*57X frameshift mutation was introduced. Male WT mice (*n* = 15) and male KO mice (*n* = 15) with a body weight range of 19–30 g were used for experiments. The mice were housed in groups of 7–8 in open Makrolon cages (37 cm × 21 cm × 14 cm) under strictly controlled laboratory conditions (temperature maintained at 21–24 °C, relative humidity at 45–65%) with an artificial 12/12 h light/dark regime (light on at 6:00 a.m.). The diet (Altromin standard diet, Altromin, Lage, Germany) and tap water were provided ad libitum. The environment was enriched with nest material and paper tubes.

The animals were randomly assigned to experimental groups. All efforts were made to minimize animal suffering as well as the number of animals used in the study. All procedures began after at least one week of acclimatization, and were performed between 8:00 a.m. and 3:00 p.m., after a minimum 30 min adaptation period to the conditions in the experimental room. The mice were closely followed-up by the animal caretakers and researchers, with regular inspection by a veterinarian, according to the standard health and animal welfare procedures of the local animal facility. With the exception of 1% ophthalmic solution of tetracaine, which was used for short-term topical ophthalmic anesthesia before acute seizure models, no anesthetics or analgesics were used, so as to reduce the possibility of a pharmacodynamic or pharmacokinetic interaction between these agents and the examined compounds. Only male mice were used, to exclude the potential impact of the estrous cycle on seizure susceptibility [38]. To avoid the possible impact of age on the seizure threshold [39], young mice were used in models of acute seizures. Each mouse was used only once in the acute seizure models.

### 2.2. Drugs

TC-G 1008, a synthetic GPR39 agonist, was purchased from AdooQ Bioscience LLC (Irvine, CA, USA). VPA (sodium salt), ZnCl_2_, and PTZ were purchased from Sigma-Aldrich. ZnCl_2_, VPA and PTZ were dissolved in physiological saline (0.9% sodium chloride solution). TC-G 1008 was suspended in 1% Tween 80 in physiological saline. Drug solutions/suspensions were prepared freshly and administered i.p. at a volume of 0.1 mL per 10 g of body weight. Control groups received vehicle (VEH), i.e., 1% Tween 80 in physiological saline. The drugs or VEH were administered 30 min before models of acute seizures, or before PTZ injection in the PTZ kindling model. This pretreatment time was chosen based on a pharmacokinetic analysis of serum and brain concentrations of TC-G 1008 after i.p. administration of this compound in Swiss albino mice [11].

### 2.3. Maximal Electroshock Seizure Model

Swiss albino mice were injected i.p. with a single dose of TC-G 1008, ZnCl_2_, VPA, or VEH; 30 min later the mice were stimulated with a supramaximal MES stimulus at a fixed current intensity (50 mA), which induced generalized tonic–clonic seizures [38]. Non-stimulated (sham) animals received the respective doses of drugs or VEH but did not receive MES stimulus.

A 1% ophthalmic solution of tetracaine was administered for short-term topical ophthalmic anesthesia. Then, constant-current stimuli (sine wave pulses at 50 Hz for 200 ms) were applied via saline-soaked transcorneal electrodes with the use of a rodent shocker (type 221; Hugo Sachs Elektronik, Freiburg, Germany). During stimulation, mice were restrained manually, and immediately following stimulation they were placed in a transparent box without bedding for behavioral observation in the presence or absence of seizure activity. Tonic hindlimb extension, defined as the rigid extension of the hindlimb exceeding a 90° angle with the body, was considered as an endpoint.

The doses of drugs administered in this model were selected based on the behavioral outcomes of the MEST test, which was conducted previously [11]. Briefly, in the MEST test, an initial dose of the examined drug was chosen, and the dose was either increased or decreased in a subsequent group of mice, depending on whether the previous dose affected the seizure threshold. We found dose-dependent effects of TC-G 1008 and ZnCl_2_ in the MEST test. The 2.5 mg/kg dose of TC-G 1008 did not significantly affect the seizure threshold, whereas doses of 5, 10, and 20 mg/kg decreased the seizure threshold in this test. The dose of ZnCl_2_ corresponding to 4 mg of Zn/kg did not affect the seizure threshold, whereas the doses of 8 and 16 mg of Zn/kg decreased the seizure threshold in this test [11]. Here, we used ineffective (2.5 mg/kg) or effective (20 mg/kg) doses of TC-G 1008. Similarly, we used ineffective (4 mg Zn/kg) or effective (16 mg Zn/kg) doses of Zn. The dose of VPA of 150 mg/kg is commonly used in this paradigm [38], and has been established to increase the seizure threshold in this test [40].

### 2.4. Six Hertz (6 Hz) Seizure Model

Swiss albino mice were injected with a single dose of TC-G 1008, ZnCl_2_, VPA, or VEH; 30 min later the mice were stimulated with a supramaximal 6 Hz stimulus at a fixed current intensity (32 mA), which induced focal seizures [41]. Non-stimulated (sham) animals received the respective doses of drugs or VEH but did not receive the 6 Hz stimulus.

A 1% ophthalmic solution of tetracaine was used for short-term topical ophthalmic anesthesia. Then, square-wave alternating current stimuli (0.2 ms duration pulses at 6 Hz for 3 s) were applied via saline-soaked transcorneal electrodes using a Grass model CCU1 constant-current unit coupled with a Grass S48 stimulator (Grass Technologies, Warwick, RI, USA). The mice were manually restrained during the stimulation. Immediately following the stimulation, mice were placed in a transparent box without bedding for behavioral observation. The 6 Hz seizures were characterized by stun (fixed) posture, rearing, forelimb clonus, twitching of the vibrissae, and elevated tail.

The doses of drugs administered in this model were selected based on the behavioral outcomes of the 6-hertz-induced seizure threshold test, which was conducted previously [11]. Briefly, in the 6 Hz seizure threshold test, an initial dose of the examined drug was chosen, and the dose was either increased or decreased in a subsequent group of mice, depending on whether the previous dose affected the seizure threshold. We found dose-dependent effects of TC-G 1008 and ZnCl_2_ in the 6 Hz seizure threshold test. TC-G 1008 at doses of 40 and 20 mg/kg increased the threshold for seizures in this test, while the dose of 10 mg/kg was ineffective. ZnCl_2_ at doses of 8 and 4 mg of Zn/kg increased the threshold for seizure in the 6 Hz seizure threshold test, while the dose of 1 mg of Zn/kg was ineffective [11]. In this study, we used doses that were effective in this test, i.e., TC-G 1008 40 mg/kg and Zn 8 mg/kg. The 50 mg/kg dose of VPA is commonly used in this paradigm and has been established to increase the seizure threshold in this test [40].

### 2.5. PTZ Kindling Model

The PTZ-induced kindling model of epilepsy is a chronic rodent model for studying the process of epilepsy’s development, i.e., epileptogenesis. In this model, epileptogenesis is triggered by repeated administration of the chemical substance PTZ—a GABA_A_ receptor antagonist. The main feature of PTZ-kindling is the progressive increase in seizure severity or duration with repeated exposure to a subthreshold dose of PTZ [42].

The PTZ kindling model was performed in Swiss albino or C57BL/6 × CBA *Gpr39*-KO and WT mice. The mice were injected i.p. with TC-G 1008, ZnCl_2_, VPA, or VEH on every alternate day during weekdays; 30 min later, kindled mice were injected i.p. with a subthreshold dose of PTZ. In the case of kindling in Swiss albino mice, the subthreshold dose of PTZ was 40 mg/kg [43]. In the case of kindling in C57BL/6/Tar × CBA/Tar mice, the subthreshold dose of PTZ was 25 mg/kg [11]. Non-kindled mice received the respective doses of drugs or VEH, but were injected with physiological saline instead of PTZ solution.

The protocols for the aforementioned models and the behavioral outcomes were previously described [11]. Briefly, immediately following PTZ injection, mice were placed individually into a transparent box without bedding for 30 min for behavioral observation. The seizure severity of each subject was scored using the modified Racine’s scale: stage 0, no response; stage 1, immobility, ear and facial twitching; stage 2, myoclonic jerks; stage 3, forelimb clonus; stage 4, clonic seizure with rearing and falling; stage 5, generalized clonic seizure with loss of righting reflex; stage 6, tonic fore- and hindlimb extension [43]. The mean seizure severity scores were calculated for all experimental groups after each PTZ injection. Mice were considered fully kindled when they displayed consecutive stage 5 or 6 seizures.

The kindling model in Swiss albino mice consisted of 19 injections of PTZ (40 mg/kg). After 19 injections of PTZ, the percentages of fully kindled Swiss albino mice were 6.7% of mice treated with VPA (150 mg/kg), 53% of mice treated with VEH, 67% of mice treated with ZnCl_2_ (8 mg Zn/kg), and 87% of mice treated with TC-G 1008 (10 mg/kg). The kindling model in C57BL/6/Tar × CBA/Tar mice consisted of 14 injections of PTZ (25 mg/kg). After 14 injections of PTZ, the percentages of fully kindled mice were 25% of the WT VEH group, 83.3% of the WT TC-G 1008 (10 mg/kg) group, 0% of the *Gpr39*-KO VEH group, and 0% of the *Gpr39*-KO TC-G 1008 (10 mg/kg) group. Thus, the percentages of fully kindled Swiss albino or WT (C57BL/6/Tar × CBA/Tar) mice that received TC-G 1008 were similar (>80%) at the end of both kindling procedures [11]. To exclude the possible impact of age on the maximal seizure severity [44], the age of mice used in both kindling procedures (Swiss albino mice or C57BL/6 × CBA mice) was similar at the beginning of these paradigms.

### 2.6. Tissue Processing for Biochemical Analysis

The mice were killed ca. 3 min after acute MES or 6 Hz seizures, or 24 h after the completion of the chronic kindling paradigms. The brains were rapidly dissected and immersed in cooled (2–8 °C) 0.9% NaCl solution. The brains were rapidly dissected on a cold plate into the left and right hemispheres. Left hippocampi (dorsal and ventral) were dissected, immediately frozen on dry ice, and stored at −80 °C until Western blot analysis. The right hemispheres were frozen with liquid nitrogen and stored at −80 °C until cryosectioning.

The trunk blood was collected into tubes without anticoagulant. The blood was allowed to clot for 15–20 min and then centrifuged for 10 min at 5600 rpm at 4 °C. The resulting supernatant (serum) was pipetted into tubes that were stored at −80 °C until ICP-OES analysis.

### 2.7. Inductively Coupled Plasma Optical Emission Spectrometry (ICP-OES)

The serum samples were defrosted; 200 µL of serum was transferred to digestion vessels (DigiTUBE SCP SCIENCE 50 mL class A) and mixed with 1.5 mL of 65% Suprapur^®^ nitric acid (Merck, Darmstadt, Germany) and 5.0 mL of deionized water. Then, vessels were placed in heating blocks (DigiPREP SCP SCIENCE) and digested for 60 min at 120 °C. After digestion, vessels with solution were left to reach room temperature (RT) and were filled with deionized water to 10 mL. The analysis was performed using PlasmaQuant PQ 9000 Analytik Jena AG. The following operating conditions of ICP-OES were used: power 13,000 W, plasma gas 14.0 L/min, auxiliary gas 0.50 L/min, nebulizer gas 0.60 L/min, and the monitoring direction of the plasma flame was axial. Standard solution for calibration curves of magnesium at the concentration of 1000 µg/L was prepared by diluting magnesium 1000 mg/L standard (PlasmaCAL SCP SCIENCE) with 0.5% nitric acid in deionized water. The analysis line used for magnesium quantification was 279.08 nm. Each serum sample was run in triplicate.

### 2.8. Laser Ablation Inductively Coupled Plasma Mass Spectrometry (LA-ICP-MS)

First, 12 µm hippocampal coronal sections were prepared using a Leica CM 1850 cryostat microtome (Germany), and were attached to glass slides (SuperFrost microscope slides, cut edges, Thermo Scientific Menzel Glaser). The glass slides were stored at −80 °C until LA-ICP-MS analysis. The sections on glass slides were thawed and dried at RT in the desiccator and placed in the ablation chamber. The sections were analyzed using a laser ablation (LA) system (LSX-500, CETAC Technologies, Omaha, NE, USA) with a quadrupole inductively coupled plasma mass spectrometer (ICP-MS, Elan DRC II, PerkinElmer SCIEX, Toronto, ON, Canada). The instruments were optimized daily with the use of a certified reference material of NIST SRM 610 glass, which included the nebulizer gas flow, ion lens voltage, and power of the plasma generator, and were tuned until reaching the maximal intensity for 24Mg+, 115In+, 238U+, and the oxide ratios of 232Th16O+/232Th+ < 0.2%, as well as doubly charged ions 42Ca2+/42Ca+ < 0.2%. The following isotopes were monitored in all measurements: 13C, 26Mg. The laser parameters were optimized to completely ablate the thin sections and to obtain measurable signals for the analyzed elements, which required the optimization of the following laser parameters: laser energy, laser spot size, frequency of laser shots, and sample scan rate. The instrumental parameters of LA-ICP-MS were as follows: laser energy 2.7 mJ, spot size 100 µm, ablation frequency 2 Hz, scan rate 100 µm/s, nebulizer gas flow 0.9 L/min, plasma power 1250 W, pulse counting mode, dwell time 100 ms per isotope, measured isotopes 13C and 26Mg. The laser beam scanned a rectangular area of the sample line by line and always from left to right. The number and width of the ablation lines were set individually for each analyzed sample, with 18 lines on average. The detector recorded a time-resolved signal that was used to create a two-dimensional matrix of data points for each sample. For the statistical evaluation of the measurement data, the region of interest (ROI) containing the hippocampus was marked on maps of the distribution of elements in thin sections of brain. The ROIs were selected by drawing the shape in the imaging software tool based on the photograph of the sample taken prior to the ablation. The signals contained in the ROI were averaged and evaluated statistically. Each sample was run in at least triplicate. In total, 51 sections were ablated: 13 sections in the case of the WT VEH group, 12 sections in the case of the *Gpr39*-KO VEH group, 14 sections in the case of the WT TC-G 1008 group, and 12 sections in the case of the *Gpr39*-KO TC-G 1008 group.

### 2.9. Western Blot

Hippocampi were homogenized in 2% sodium dodecyl sulfate solution (SDS) (BioShop Canada Inc.), denatured at 95 °C for 10 min, and centrifuged at 10,000 rpm at 4 °C for 5 min. The total protein concentration was quantified in the supernatant using a Pierce BCA Protein Assay Kit (Thermo Fisher Scientific, Pierce Biotechnology, Rockford, IL, USA). The samples containing 10 µg of protein were prepared using Novex^®^ Tris-Glycine SDS Sample Buffer (Thermo Fisher Scientific, Carlsbad, CA, USA), and were resolved on Mini-Protean TGX Precast gels (BIO-RAD Laboratories, Inc., Hercules, CA, USA). The molecular weight marker Precision Plus Dual Color Standard (BIO-RAD Laboratories, Inc., USA) was used. The proteins were transferred on a nitrocellulose membrane (BIO-RAD Laboratories, Inc., USA). The membranes were blocked for 60 min with 1% blocking reagent from the BM Chemiluminescence WB Kit (Mouse/Rabbit) (Roche Diagnostic, Mannheim, Germany). The membranes were then incubated with rabbit polyclonal antibody targeting TRPM7 (Novus Biologicals Cat# NBP2-20739, at a dilution of 1:3000), rabbit polyclonal antibody targeting SLC41A1 (Novus Biologicals Cat# NBP1-82652, at a concentration of 0.4 µg/mL), or β-actin (Sigma-Aldrich Cat# A1978, at a concentration of 0.5 µg/mL) at 2–8 °C overnight. The dilutions of primary antibodies were prepared using 0.5% blocking solution from the BM Chemiluminescence WB Kit (Mouse/Rabbit). They were stored at 2–8 °C and were reused up to three times. On the next day, after washing with TBST 3 × 10 min, the membranes were incubated for 30 min with horseradish-peroxidase-linked (HRP-linked) secondary antibody from the BM Chemiluminescence WB Kit (Mouse/Rabbit), or the anti-mouse IgG, HRP-linked, Cell Signaling Cat# 7076, at a dilution of 1:1000 under constant shaking at RT. The dilutions of secondary antibodies were always prepared fresh. After incubation with secondary antibodies, the membranes were washed with TBST 3 × 10 min. Secondary antibodies were detected using a BM Chemiluminescence WB Kit (Mouse/Rabbit). The protein bands were visualized with the ChemiDoc Imaging System (BIO-RAD Laboratories, Inc., USA). The density of each protein band was analyzed using imaging software (Image Lab, BIO-RAD Laboratories, Inc., USA), and was normalized by the optical density of the corresponding β-actin band. Each sample was run in triplicate.

### 2.10. Data and Statistical Analysis

Data were analyzed using GraphPad Prism v. 9 (GraphPad Software, San Diego, CA, USA) or STATISTICA v. 13.3 (TIBCO Software Inc., Palo Alto, CA, USA) by two-way or the three-way ANOVA and Tukey’s/Bonferroni’s multiple comparison test. All results are presented as the mean ± SEM; *p* < 0.05 was considered statistically significant with 95% confidence. No statistical method was used to predetermine sample size. The exact sample size is shown in figure legends or on graphs. Data were screened for outliers using Grubbs’ test (https://www.graphpad.com/quickcalcs/Grubbs1.cfm, accessed on 1 January 2022), and outliers were excluded from the analyses. The analyses were performed by experimenters blinded to the treatment.

## 3. Results

### 3.1. The Effects of Acute Treatment with TC-G 1008 and MES or 6 Hz Seizures on Total Serum Magnesium Concentration

**Figure 1:**
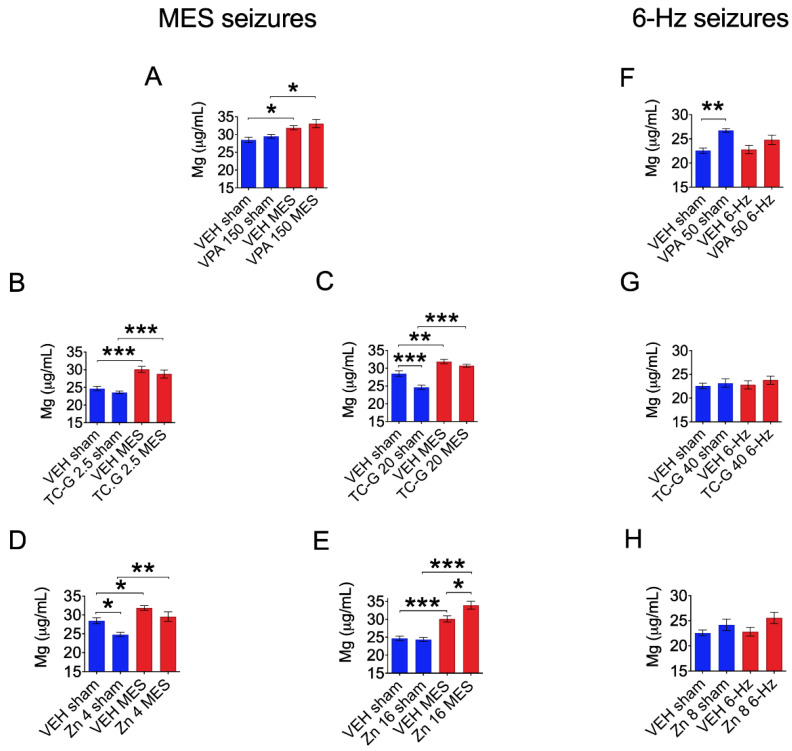
The effects of acute treatment with valproic acid (VPA) (**A**,**F**), TC-G 1008 (**B**,**C**,**G**), or ZnCl_2_ (**D**,**E**,**H**) and maximal electroshock (MES) seizures (**A**–**E**) or the 6 Hz (**F**–**H**) seizures on total serum magnesium concentrations. Drugs (the doses are shown on the abscissas) or vehicle (VEH) (1% Tween 80 in physiological saline) were administered i.p., 30 min before MES or 6 Hz seizures, in Swiss albino mice. Non-stimulated (sham) animals received the same doses of drugs or VEH, but they did not receive the electrical stimulus. Total serum magnesium concentration was analyzed by inductively coupled plasma optical emission spectrometry (ICP-OES) at ca. 3 min after MES or 6 Hz seizures. Data are expressed as means ± SEM. Data were analyzed by two-way ANOVA and Tukey’s multiple comparison test. Statistical details: (**A**) Interaction [F(1,36) = 0.0111, *p* = 0.9168], VPA 150 [F(1,36) = 1.835, *p* = 0.1840], MES [F(1,36) = 18.48, *p* = 0.0001]; *n* = 10 in each group (**B**) Interaction [F(1,30) = 0.0144, *p* = 0.9051], TC-G 1008 2.5 [F(1,30) = 2.611, *p* = 0.1166], MES [F(1,30) = 50.66, *p* < 0.0001]; *n* = 10 VEH sham, *n* = 8 VEH MES, *n* = 9 TC-G 1008 2.5 sham, *n* = 7 TC-G 1008 2.5 MES (**C**) Interaction [F(1,34) = 7.875, *p* = 0.0082], TC-G 1008 20 [F(1,34) = 22.19, *p* < 0.0001], MES [F(1,34) = 71.73, *p* < 0.0001]; *n* = 10 in each group (**D**) Interaction [F(1,33) = 6.067, *p* = 0.0192], Zn 4 [F(1,33) = 7.367, *p* = 0.0105], MES [F(1,33) = 51.75, *p* < 0.0001]; *n* = 10 in each group (**E**) Interaction [F(1,33) = 6.925, *p* = 0.0128], Zn 16 [F(1,33) = 4.86, *p* = 0.0346], MES [F(1,33) = 89.11, *p* < 0.0001]; *n* = 10 VEH sham, *n* = 8 VEH MES, *n* = 10 Zn 16 sham, *n* = 9 Zn 16 MES (**F**) Interaction [F(1,27) = 2.03, *p* = 0.1657], VPA 50 [F(1,27) = 16.41, *p* = 0.0004], 6-Hz seizure [F(1,27) = 1.231, *p* = 0.277]; *n* = 8 in each group, 1 outlier was detected in VPA 50 sham group (**G**) Interaction [F(1,28) = 0.0660, *p* = 0.799], TC-G 1008 40 [F(1,28) = 0.9687, *p* = 0.3334], 6-Hz seizure [F(1,28) = 0.3113, *p* = 0.5813]; *n* = 8 in each group (**H**) Interaction [F(1,28) = 0.3783, *p* = 0.5435], Zn 8 [F(1,28) = 5.23, *p* = 0.03], 6-Hz [F(1,28) = 0.7524, *p* = 0.3931], *n* = 8 in each group (by two-way ANOVA). * *p* < 0.05, ** *p* < 0.01, *** *p* < 0.001 (by Tukey’s multiple comparison test).

We examined total serum magnesium concentrations in Swiss albino mice subjected to models of acute seizures (MES or 6 Hz seizures) or a chronic PTZ-induced kindling model of epilepsy. MES seizures, unlike 6 Hz seizures, significantly increased total serum magnesium concentrations (Figure 1A–H). Furthermore, serum magnesium concentrations were increased in mice that received VPA (Figure 1A)TC-G 1008 (Figure 1B,C) or ZnCl_2_ (Figure 1D,E), and MES, compared to sham-treated mice that received the same drugs. In addition, administration of TC-G 1008 (20 mg/kg) decreased serum magnesium concentrations in sham-treated mice (Figure 1C). Furthermore, administration of Zn (4 mg Zn/kg) decreased serum magnesium concentrations in sham-treated mice (Figure 1D). Conversely, administration of Zn (16 mg Zn/kg) increased serum magnesium concentrations in MES-treated mice (Figure 1E). Acute treatment with VPA (50 mg/kg) increased serum magnesium concentration in sham-treated mice (Figure 1F).

### 3.2. The Effects of Chronic Treatment with TC-G 1008 in Mice Subjected to the PTZ Kindling Model of Epilepsy on Total Serum Magnesium Concentration

**Figure 2:**
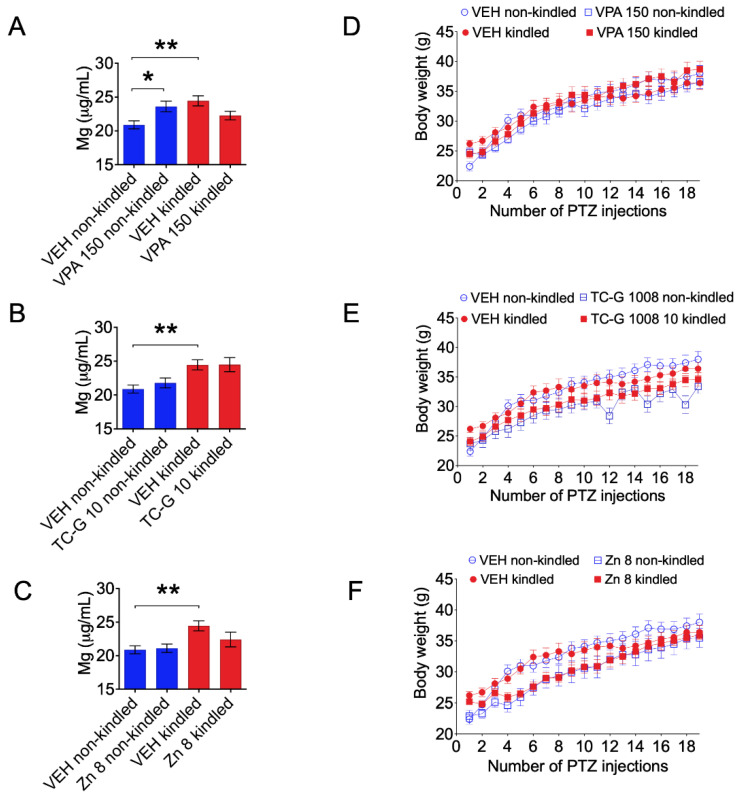
(**A**–**C**) The effects of chronic treatment with VPA (150 mg/kg) (**A**), TC-G 1008 (10 mg/kg) (**B**), or ZnCl_2_ (8 mg Zn/kg) (**C**) in the pentylenetetrazole (PTZ)-induced kindling model of epilepsy on total serum magnesium concentration. Drugs or VEH (1% Tween 80 in physiological saline) were injected i.p. once daily on every alternate day during weekdays in Swiss albino mice. In kindled mice, PTZ (40 mg/kg) was injected i.p. 30 min later. Non-kindled mice received physiological saline instead of PTZ. The model consisted of 19 injections of PTZ. Total serum magnesium concentration was measured by ICP-OES at 24 h after the completion of the model. Data are expressed as means ± SEM. Data were analyzed by two-way ANOVA and Tukey’s multiple comparison test. Statistical details: (**A**) VPA [F(1,35) = 0.1492, *p* = 0.7016], PTZ kindling [F(1,35) = 2.522, *p* = 0.1213], VPA × PTZ kindling [F(1,35) = 12.29, *p* = 0.0013]; *n* = 10 VEH non-kindled, VEH kindled, VPA 150 non-kindled, *n* = 9 VPA 150 kindled (**B**) TC-G 1008 10 [F(1,35) = 0.4007, *p* = 0.5308], PTZ kindling [F(1,35) = 15.05, *p* = 0.0004], TC-G 1008 × PTZ kindling [F(1,35) = 0.3397, *p* = 0.5637]; *n* = 10 VEH non-kindled, VEH kindled, TC-G 1008 10 non-kindled, *n* = 9 TC-G 1008 10 kindled (**C**) Zn [F(1,35) = 1.376, *p* = 0.8032], PTZ kindling [F(1,35) = 9.813, *p* = 0.035], Zn × PTZ kindling [F(1,35) = 2.122, *p* = 0.1541]; *n* = 10 VEH non-kindled, VEH kindled, Zn 8 non-kindled, *n* = 9 Zn 8 kindled (by two-way ANOVA). * *p* < 0.05, ** *p* < 0.01 (by Tukey’s multiple comparison test). (**D**–**F**) The effects of chronic treatment with VPA (**D**), TC-G 1008 (**E**), or ZnCl_2_ (**F**) on the body weight of mice subjected to the PTZ kindling model. Data were analyzed by three-way repeated-measures ANOVA. Statistical details: (**D**) VPA [F(1,36) = 0.205, *p* = 0.6535], PTZ kindling [F(1,36) = 0.328, *p* = 0.570193], VPA × PTZ kindling [F(1,36) = 0.824, *p* = 0.367], time [F(18,648) = 617.8, *p* < 0.0001], time × VPA [F(18,648) = 2.229, *p* = 0.0025], time × PTZ kindling [F(18,648) = 1.153, *p* = 0.2961], time × VPA × PTZ kindling [F(18,648) = 4.504, *p* < 0.0001]; *n* = 10 in each group (**E**) TC-G 1008 [F(1,36) = 7.105, *p* = 0.0114], PTZ kindling [F(1,36) = 0.132, *p* = 0.7188], TC-G 1008 × PTZ kindling [F(1,36) = 0.467, *p* = 0.4988], time [F(18,648) = 185.682, *p* < 0.0001], time × TC-G 1008 [F(18,648) = 4.314, *p* < 0.0001], time × PTZ kindling [F(18,648) = 3.515, *p* < 0.0001], time × TC-G 1008 × PTZ kindling [F(18,648) = 5.101, *p* < 0.0001]; *n* = 10 in each group (**F**) Zn [F(1,36) = 6.028, *p* = 0.0190], PTZ kindling [F(1,36) = 0.010, *p* = 0.9218], Zn × PTZ kindling [F(1,36) = 0.179, *p* = 0.674716], time [F(18,648) = 189.353, *p* < 0.0001], time × Zn [F(18,648) = 4.540, *p* < 0.0001], time × PTZ kindling [F(18,648) = 3.315, *p* < 0.0001], time × Zn × PTZ kindling [F(18,648) = 1.557, *p* = 0.0655]; *n* = 10 in each group by three-way repeated-measures ANOVA).

Total serum magnesium concentrations were also significantly increased in kindled Swiss albino mice at 24 h after the completion of the chronic, PTZ kindling model, which consisted of 19 injections of PTZ (40 mg/kg) (Figure 2A–C). Moreover, chronic treatment with VPA (150 mg/kg) increased serum magnesium concentrations in non-kindled mice (Figure 2A).

In addition, we assessed the body weight of mice subjected to the PTZ kindling model. There was no effect of VPA on the body weight of mice, but a significant time × VPA × PTZ interaction (Figure 2D). There was a significant effect of TC-G 1008 on the body weight of mice, and a significant time × TC-G 1008 × PTZ interaction (Figure 2E). Furthermore, there was a significant effect of ZnCl_2_ on the body weight of mice (Figure 2F).

### 3.3. The Effects of Chronic Treatment with TC-G 1008 in Gpr39-KO and WT Mice Subjected to the PTZ Kindling Model of Epilepsy on Body Weight

**Figure 3:**
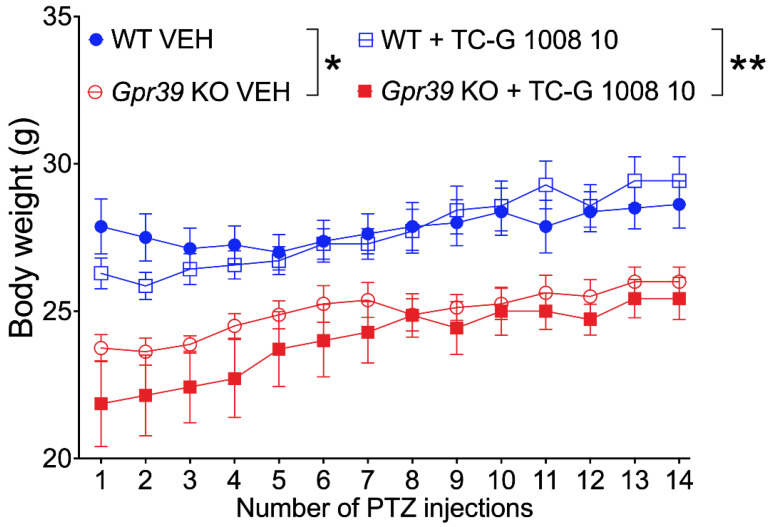
The effects of chronic treatment with TC-G 1008 (10 mg/kg) on the body weight of *Gpr39*-KO or WT (C57BL/6/Tar × CBA/Tar) mice subjected to the PTZ kindling model. The mice were injected i.p. with TC-G 1008 or VEH (1% Tween 80 in physiological saline) on every alternate day during weekdays; 30 min later, PTZ (25 mg/kg) was injected i.p. The model consisted of 14 injections of PTZ. Data were analyzed by three-way ANOVA and Bonferroni’s multiple comparison test. Statistical details: genotype [F(1,26) = 22.743, *p* < 0.0001]; drug [F(1,26) = 0.682, *p* = 0.416334]; genotype × drug [F(1,26) = 0.335, *p* = 0.567442]; time [F(13,338) = 32.006, *p* < 0.0001]; time × genotype [F(13,338) = 4.671, *p* = 0.0353]; time × drug [F(13,338) = 1.847, *p* < 0.0001] (by three-way repeated measures ANOVA); *n* = 8 WT VEH, *n* = 7 WT TC-G 1008, *n* = 8 KO VEH, *n* = 7 KO TC-G 1008. * *p* < 0.05, ** *p* < 0.01 (by Bonferroni’s multiple comparison test).

We then assessed the body weight of *Gpr39*-KO and WT C57BL/6/Tar × CBA/Tar mice subjected to the PTZ kindling model. There was a significant effect of genotype on the body weight of C57BL/6/Tar × CBA/Tar mice. The body weight of *Gpr39*-KO mice that received VEH was lower compared to WT mice. Moreover, the body weight of *Gpr39*-KO mice that received TC-G 1008 during PTZ kindling was lower compared to WT mice that received this compound during this procedure (Figure 3).

### 3.4. The Effects of Chronic Treatment with TC-G 1008 in Gpr39-KO and WT Mice Subjected to the PTZ Kindling Model of Epilepsy on Total Serum Magnesium Concentration

**Figure 4:**
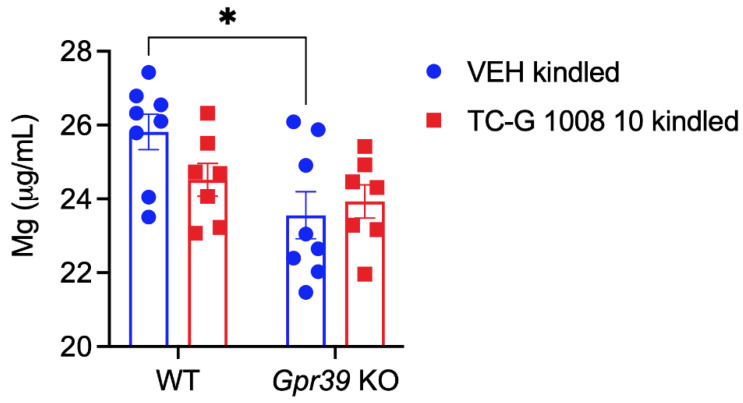
The effects of the PTZ kindling model and chronic treatment with TC-G 1008 (10 mg/kg) in *Gpr39*-KO or WT (C57BL/6/Tar × CBA/Tar) mice on total serum magnesium concentration. ICP-OES analysis was performed at 24 h after the completion of the model. Data are expressed as means ± SEM. Data were analyzed by two-way ANOVA and Tukey’s multiple comparison test. Statistical details: genotype effect [F(1,26) = 7.526, *p* = 0.0109]; drug effect [F(1,26) = 0.8014, *p* = 0.3789]; genotype × drug effect [F(1,26) = 2.584, *p* = 0.1200] (by two-way ANOVA).* *p* < 0.05 (by Tukey’s multiple comparison test).

We next evaluated total serum magnesium concentrations in *Gpr39*-KO and WT C57BL/6/Tar × CBA/Tar mice subjected to the PTZ kindling model. There was a significant effect of genotype on serum magnesium concentration at 24 h after the completion of the kindling paradigm, which consisted of 14 injections of PTZ (25 mg/kg). Serum magnesium concentration was decreased in *Gpr39*-KO mice that received VEH during this model, compared to WT mice that underwent the kindling procedure (Figure 4).

### 3.5. The Effects of Chronic Treatment with TC-G 1008 in Gpr39-KO and WT Mice Subjected to the PTZ Kindling Model of Epilepsy on the Expression of the TRPM7 and SLC41A1 Proteins in the Hippocampus

**Figure 5:**
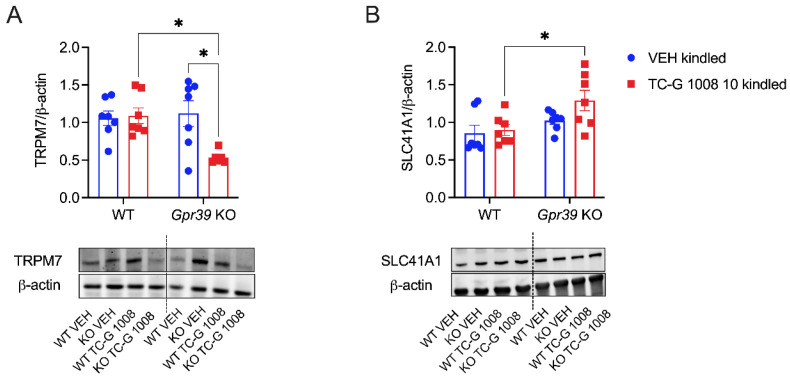
The effects of the PTZ kindling model and chronic treatment with TC-G 1008 (10 mg/kg i.p.) in *Gpr39*-KO or WT (C57BL/6/Tar × CBA/Tar) mice on the expression of the proteins TRPM7 (**A**) and SLC41A1 (**B**) in the hippocampus. Protein expression was examined at 24 h after the completion of the model. Data are expressed as means ± SEM. Data were analyzed by two-way ANOVA and Tukey’s multiple comparison test. Statistical details: (**A**) genotype effect [F(1,23) = 4.411, *p* = 0.0469]; drug effect [F(1,23) = 5.521, *p* = 0.0277]; genotype × drug effect [F(1,23) = 6.937, *p* = 0.0148] (**B**) genotype effect [F(1,24) = 8.407, *p* = 0.0079]; drug effect [F(1,24) = 2.592, *p* = 0.1205]; genotype × drug effect [F(1,24) = 1.358, *p* = 0.2554] (by two-way ANOVA). * *p* < 0.05 (by Tukey’s multiple comparison test).

Furthermore, we examined the expression of proteins that are associated with magnesium transport into and out of cells, i.e., TRPM7 and SLC41A1, respectively, in the hippocampi of *Gpr39*-KO and WT C57BL/6/Tar × CBA/Tar mice subjected to the PTZ kindling model. There was a significant effect of genotype on the expression of both proteins at 24 h after the completion of the kindling paradigm. In addition, there was a significant effect of drug on the expression of TRPM7 protein, and a genotype × drug interaction. The levels of protein expression of TRPM7 (Figure 5A) or SLC41A1 (Figure 5B) did not differ significantly between *Gpr39*-KO and WT mice that received VEH during the model. Surprisingly, treatment with TC-G 1008 decreased the level of expression of TRPM7 in the hippocampi of *Gpr39*-KO mice. Moreover, the expression of this protein was lower in the hippocampi of *Gpr39*-KO mice treated with TC-G 1008, compared to WT mice treated with the same compound (Figure 5A). Decreased levels of TRPM7 were accompanied by increased levels of SLC41A1. The expression of the latter protein was higher in the hippocampi of *Gpr39*-KO mice treated with TC-G 1008, compared to WT mice treated with the same compound (Figure 5B).

### 3.6. The Effects of Chronic Treatment with TC-G 1008 in Gpr39-KO and WT Mice Subjected to the PTZ Kindling Model of Epilepsy on Total Magnesium in the Hippocampus

**Figure 6:**
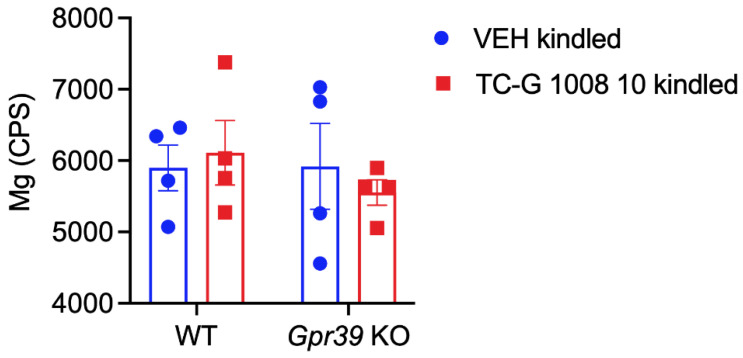
The effects of the PTZ kindling model and chronic treatment with TC-G 1008 (10 mg/kg i.p.) in *Gpr39*-KO or WT (C57BL/6/Tar × CBA/Tar) mice on total magnesium in the hippocampus. Laser ablation inductively coupled plasma mass spectrometry (LA-ICP-MS) analysis was performed at 24 h after the completion of the model. Results are presented as counts per second (CPS). Data are expressed as means ± SEM. Data were analyzed by two-way ANOVA. Statistical details: genotype effect [F(1,12) = 0.4079, *p* = 0.5351]; drug effect [F(1,12) = 0.03297, *p* = 0.8590]; genotype × drug effect [F(1,12) = 0.4762, *p* = 0.5033].

Finally, we assessed total magnesium content of the hippocampus in *Gpr39*-KO and WT C57BL/6/Tar × CBA/Tar mice subjected to the PTZ kindling model. The LA-ICP-MS method did not reveal statistically significant differences in total magnesium levels in the hippocampus between *Gpr39*-KO or WT mice subjected to this model, that received either VEH or TC-G 1008 during the kindling procedure (Figure 6).

## 4. Discussion

In this study, we found increased serum magnesium concentrations in mice subjected to the MES model and PTZ kindling model, and decreased serum magnesium concentrations in *Gpr39*-KO mice subjected to the latter model. Decreased serum magnesium concentrations in the sera of *Gpr39*-KO C57BL/6 × CBA mice led us to investigate whether there were corresponding changes in the brain. Using the methodology presented here (i.e., LA-ICP-MS), we did not, however, demonstrate corresponding changes in the hippocampal tissue. There are several possible explanations for these findings. The LA-ICP-MS method, similarly to ICP-OES, detects total levels of an element, i.e., the form bound to proteins and other ligands as well as the ionic form. The changes in magnesium concentration observed in the present study in sera were mild, but they were detectable using ICP-OES. The techniques detecting total elemental content in blood/serum show a lack of sensitivity towards moderate changes induced, e.g., by a diet [45,46]. Hence, even though the alterations found in sera were mild, they were detectable using the method for assessing the total pool of magnesium, thus suggesting the interference of the *Gpr39*-KO genotype with proteins and other ligands to which magnesium is bound.

The lack of differences in magnesium levels in the hippocampal tissue may be due to homeostatic mechanisms tightly controlling the brain levels [47]. Similarly to inducing hypermagnesemia, which in humans does not concomitantly increase magnesium levels in the brain [48], (mild) hypomagnesemia may not affect the total brain levels of this element. Alternatively, the changes in the brain may only involve the pool of ionic magnesium and, therefore, could not have been detected by the method applied here. Several types of relationships between ionized and total magnesium measured in one tissue type or in different tissues have been described, which may be of importance in terms of neurological disorders. Decreased ionized magnesium with no concomitant changes in total magnesium was found in blood in the lateral fluid percussion model of traumatic brain injury (TBI) [49]. TBI is a risk factor for developing epilepsy. In this model of TBI, increased susceptibility to seizures was observed [50]. In newborn swine, intravenous infusions of magnesium sulfate raised total plasma magnesium concentrations, as measured by atomic absorption spectroscopy. Increased total plasma magnesium was not accompanied by changes in cerebral intracellular magnesium concentration, and no correlation between plasma and cerebral intracellular magnesium was observed [51]. Furthermore, reduced ionic magnesium was demonstrated in the occipital lobes of patients with different types of migraine and cluster headache, as measured by in vivo phosphorus magnetic resonance spectroscopy [52]. Taken together, it is plausible that in *Gpr39*-KO mice subjected to the PTZ kindling model there are no changes in the levels of protein/ligand-bound magnesium in the hippocampus, but there may be alterations in ionic magnesium, which may or may not be parallel to those observed in serum.

Changes in the expression of proteins that are associated with magnesium transport into (TRPM7) [53] and out of (SLC41A1) [54] cells in the hippocampus of C57BL/6 × CBA mice are indeed indicative of alterations in ionic magnesium in this brain region. More specifically, decreased expression of TRPM7 and increased expression of SLC41A1 in the hippocampi of *Gpr39*-KO mice after the administration of TC-G 1008 may suggest decreased intracellular ionic hippocampal magnesium in those mice. On the other hand, alterations of the expression of these proteins may be a mechanism compensating for increased intracellular ionic magnesium.

The finding that TC-G 1008 decreased the expression of the TRPM7 protein in the hippocampus of *Gpr39*-KO mice may be surprising, since TC-G 1008 is commonly used as a selective agonist of GPR39 [55]. However, literature data and our previous observations on the effects of this synthetic GPR39 agonist in the PTZ kindling model corroborate the notion that it may act non-selectively. First, TC-G 1008 may bind to the serotonin 5HT1_A_ receptor, as was demonstrated in vitro [29]. Second, in 2020, Mo et al. used siRNA silencing to study the role of GPR39 in vitro. In their study, administration of TC-G 1008 to cells in which GPR39 expression was silenced induced increased expression of CREB, compared to the siRNA-treated group [56]. Third, we previously found that TC-G 1008 markedly and significantly increased the phosphorylated CREB (p-CREB)/CREB ratio in the hippocampi of *Gpr39*-KO mice in the PTZ kindling model. Moreover, the phosphorylated tropomyosin receptor kinase B (p-TrkB)/TrkB ratio was increased (though not significantly) in the hippocampi of GPR39-KO mice treated with TC-G 1008 [11].

The p-CREB/CREB and p-TrkB/TrkB ratios were also increased in the hippocampi of GPR39-KO mice treated with TC-G 1008, when compared to WT mice that received the same treatment [11]. Regarding the behavioral outcomes of the model, administration of TC-G 1008 in *Gpr39*-KO mice did not affect the maximal seizure score. In turn, the group of WT mice treated with TC-G 1008 displayed significantly higher seizure scores, compared to WT mice without drug treatment. At the same time, the behavioral scores of the *Gpr39*-KO TC-G 1008 group were lower than those of the WT TC-G 1008 group [11]. Summarizing our previous and current data on the effects of TC-G 1008 in the PTZ kindling model, *Gpr39*-KO mice treated with TC-G 1008 displayed less pronounced seizure stages, increased activation of CREB and TrkB in the hippocampus, decreased expression of TRPM7, and increased expression of SLC41A1 in this brain region, compared to WT mice that received the same treatment (Table 1).

TRPM7 overexpression or overactivation was suggested to mediate neurodegenerative processes following triggers such as hypoxia or ischemia [21]. In terms of seizures, inhibition of TRPM7 was associated with a positive treatment effect. Carvacrol, a naturally occurring monoterpenic phenol that (among other mechanisms) inhibits TRPM7, was active in the perforant path stimulation kindling model of epilepsy, and inhibited recurrent status epilepticus [57]. Less pronounced seizure stages in the *Gpr39*-KO TC-G 1008 group may thus be related to decreased TRPM7 expression (Table 1) and, presumably, its decreased function in the hippocampus.

The involvement of the tropomyosin receptor kinase A (TrkA) pathway in the regulation of TRPM7 in hippocampal neurons subjected to ischemic reperfusion and oxygen–glucose deprivation was demonstrated [58]. More specifically, activation of TrkA by nerve growth factor (NGF) prevented upregulation of TRMP7 in hippocampal neurons [58]. This and other studies indicated the regulation of TRPM7 by receptors for growth factors [59]. Brain-derived neurotrophic factor (BDNF) is a ligand for the TrkB receptor. The precursor form of BDNF (pro-BDNF) elicits opposite functions to BDNF. Pro-BDNF increased the surface expression of TRMP7 in microglial cells from C57BL/6J mice [60]. Our previous and current data collectively show that the activation of TrkB (as measured by the ratio of phospho/total proteins) in the hippocampi of *Gpr39*-KO mice was accompanied by decreased levels of the TRPM7 protein. These data are thus suggestive of the relationship between TRPM7 and another receptor for growth factors, i.e., TrkB, which has not been studied so far in this context.

In the present study, acute MES seizures—unlike 6 Hz seizures—and chronic seizures (PTZ kindling) significantly increased serum magnesium concentrations. Both MES and 6 Hz seizures are induced by an electrical stimulus. The interplay between the frequency and duration of electrical stimulus accounts for a differential behavioral picture. In MES, a high-frequency (50 Hz), short-duration (0.2 s) electrical stimulus evokes generalized tonic–clonic seizures. The core characteristic of the 6 Hz model, induced by a low-frequency (6 Hz) and long-duration (3 s) stimulus, is the induction of negligibly convulsive seizures, with stereotyped behavior and automatisms, resembling psychomotor seizures in humans [41]. In the PTZ kindling model, a dose of PTZ, which induces focal seizures at the beginning of the paradigm, finally triggers generalized tonic–clonic seizures [42]. Based on this picture, it may be suggested that generalized tonic–clonic seizures (i.e., MES, PTZ kindling) increased serum magnesium concentrations, while focal psychomotor seizures (6-hertz-induced) did not.

Our data reporting increased serum magnesium concentrations in mice that underwent generalized tonic–clonic seizures is consistent with the findings of Doretto et al. [61]. Serum magnesium concentrations were higher in Wistar audiogenic rats (a genetically selected strain susceptible to seizures induced by sound) that exhibited tonic–clonic seizures, compared to those rats under basal conditions. Serum magnesium concentrations were also higher in non-audiogenic Wistar rats when seizures were induced by electroshock [61]. However, human data showed that postictal (within 24 h of seizure) serum and cerebrospinal fluid magnesium levels were lower compared with interictal levels (4 weeks after seizure) [62]. A reason why the data obtained by Doretto et al. [61] and our team do not translate to these human data may be the time of sample collection for elemental analysis. We obtained the serum samples ca. 3 min after MES or 6 Hz seizures, thus examining the immediate effects of seizures on serum magnesium concentrations.

Given the acute effects of the examined agents on serum magnesium concentrations in sham mice, the lack of a dose-dependent relationship should be underlined. Zinc has many mechanisms of action [63]. Non-selective activity of TC-G 1008 in vivo was suggested by our previous [11] and current data (Table 1), and is discussed above. Moreover, chronic treatment with TC-G 1008 significantly affected serum magnesium concentrations in neither kindled Swiss mice nor *Gpr39*-KO or WT (C57BL/6 × CBA) mice. These observations contradict the assumption that chronic administration of TC-G 1008 affects serum magnesium concentration via an interaction with GPR39.

In the present study, the body weight of *Gpr39*-KO mice (C57BL/6 × CBA) was lower than that of WT mice before the first injection of PTZ, and remained lower in *Gpr39*-KO mice during the kindling procedure. The *Gpr39*-KO mouse models were bred with different genetic backgrounds. Body weight was similar between *Gpr39*-KO and WT mice (C57BL/6J) at the age of 8–24 weeks [64]. Up to the age of 16 weeks (corresponding to a body weight of ca. 30 g), no differences in body weight between *Gpr39*-KO (Sv129-C57BL/6) and WT mice were found, but higher body weight was observed in older mice (at the age of 22–81 weeks) [65]. The differences in the body weight of *Gpr39*-KO vs. WT mice may have been due to the genetic background or the diet.

Summarizing, our previous [11] and current data show that the GPR39 receptor participates in mediating the serum concentrations of zinc and magnesium. The direct link between these findings and other characteristics of *Gpr39*-KO mice remains to be established, but it is plausible, given the role of zinc and magnesium in disease areas [13,14,15,16,17,18,19] in which GPR39 is increasingly investigated (e.g., neurovascular diseases, cardiovascular diseases, bone diseases, CNS diseases such as depression, epilepsy, etc. [6,7,8,9,10,11,12]).

Using the ICP-MS method we did not find alterations in magnesium levels in the hippocampus—a region of the brain that is important in terms of epileptogenesis. Our data do not support the hypothesis that GPR39 mediates total magnesium levels in the hippocampus in the PTZ kindling model of epilepsy. However, we found that in the absence of the GPR39 receptor, TC-G 1008 influenced the expression of magnesium transporters. Changes in protein expression of TRPM7 and SLC41A in the hippocampi of GPR39-KO mice treated with TC-G 1008 thus implicitly indicate the link between this receptor and magnesium homeostasis in the hippocampus in this model.

## Figures and Tables

**Table 1:** Summary of previous and current findings on the effects of TC-G 1008 in the PTZ kindling model in *Gpr39*-KO mice. NS—not significant; VEH—vehicle (1% Tween 80 in physiological saline). The Table partly contains data from [11].

	*Gpr39*-KO vs. WT	*Gpr39*-KO TC-G 1008 vs. *Gpr39*-KO VEH	*Gpr39*-KO TC-G 1008 vs. WT TC-G 1008
**Behavioral seizures (PTZ kindling model)**	NS	NS	Decreased
**Expression of proteins in the hippocampus**			
p-CREB/CREB	NS	Increased	Increased
p-TrkB/TrkB	NS	NS (tendency towards increased)	Increased
TRMP7	NS	Decreased	Decreased
SLC41A1	NS	NS	Increased

## Data Availability

The data that support the findings of this study are available from the corresponding author upon reasonable request.
